# Supplementary material for: Non-destructive transcriptomics via vesicular export
Source: Nat Commun. 2026 Apr 25;17:3812. doi: 10.1038/s41467-026-72072-w (PMC13121718; doi:10.1038/s41467-026-72072-w)
Supplement: Supplementary file 2 — Description of Additional Supplementary Information [file 41467_2026_72072_MOESM2_ESM.pdf]

Description of Additional Supplementary Files

File Name: Supplementary Movie 1
Description: Spontaneous beating of hiPSC-derived cardiomyocytes. Representative recording at day 10 of differentiation. Cells were seeded at 800,000 cells per well in a 12-well plate. The video was acquired at 60× magnification using a Zeiss LSM 800 microscope.

File Name: Supplementary Data 1
Description: Non-overlapping genes detected in lysate but not NTVE, from the STAR-based alignment (Supplementary Figure 5b) and the minimap2-based alignment (Supplementary Figure 10c).

File Name: Supplementary Data 2
Description: Differentially expressed Genes due to forskolin treatment of murine neurons, including non-budding control and DMSO vehicle control. Relates to Figure 4.

File Name: Supplementary Data 3
Description: GSEA analyses of cardiomyocyte differentiation. Relates to Figure 7.

File Name: Supplementary Data 4
Description: NTVE constructs, AAVs, sequences, auxiliary sequences, RT-qPCR primers, pegRNAs, NGS barcodes, and NGS oligos.
