# Supplementary material for: Non-destructive transcriptomics via vesicular export
Source: Nat Commun. 2026 Apr 25;17:3812. doi: 10.1038/s41467-026-72072-w (PMC13121718; doi:10.1038/s41467-026-72072-w)
Supplement: Supplementary file 8 — Reporting Summary [file 41467_2026_72072_MOESM8_ESM.pdf]

nature portfolio

Corresponding author(s): Prof. Westmeyer, Dr. Truong

Last updated by author(s): 2026/04/01

Reporting Summary

Nature Portfolio wishes to improve the reproducibility of the work that we publish. This form provides structure for consistency and transparency in reporting. For further information on Nature Portfolio policies, see our Editorial Policies and the Editorial Policy Checklist.

Statistics

For all statistical analyses, confirm that the following items are present in the figure legend, table legend, main text, or Methods section.

n/a	Confirmed	
☐	☒	The exact sample size (n) for each experimental group/condition, given as a discrete number and unit of measurement
☐	☒	A statement on whether measurements were taken from distinct samples or whether the same sample was measured repeatedly
☐	☒	The statistical test(s) used AND whether they are one- or two-sided<br>*Only common tests should be described solely by name; describe more complex techniques in the Methods section.*
☒	☐	A description of all covariates tested
☐	☒	A description of any assumptions or corrections, such as tests of normality and adjustment for multiple comparisons
☐	☒	A full description of the statistical parameters including central tendency (e.g. means) or other basic estimates (e.g. regression coefficient) AND variation (e.g. standard deviation) or associated estimates of uncertainty (e.g. confidence intervals)
☐	☒	For null hypothesis testing, the test statistic (e.g. F, t, r) with confidence intervals, effect sizes, degrees of freedom and P value noted<br>*Give P values as exact values whenever suitable.*
☒	☐	For Bayesian analysis, information on the choice of priors and Markov chain Monte Carlo settings
☐	☒	For hierarchical and complex designs, identification of the appropriate level for tests and full reporting of outcomes
☐	☒	Estimates of effect sizes (e.g. Cohen's d, Pearson's r), indicating how they were calculated

Our web collection on statistics for biologists contains articles on many of the points above.

Software and code

Policy information about availability of computer code

Data collection	Applied Biosystems QuantStudio, BD FACS Diva
Data analysis	Python, R, fgsea, Salmon, STAR, MiniMap2, SamTools, FastQC, QuantStudio D&A, Prism10

For manuscripts utilizing custom algorithms or software that are central to the research but not yet described in published literature, software must be made available to editors and reviewers. We strongly encourage code deposition in a community repository (e.g. GitHub). See the Nature Portfolio guidelines for submitting code & software for further information.

Data

Policy information about availability of data

All manuscripts must include a data availability statement. This statement should provide the following information, where applicable:

- Accession codes, unique identifiers, or web links for publicly available datasets
- A description of any restrictions on data availability
- For clinical datasets or third party data, please ensure that the statement adheres to our policy

The RNA sequencing data generated in this study have been deposited in the Gene Expression Omnibus (GEO) database under accession code GSE283136 [https://www.ncbi.nlm.nih.gov/geo/query/acc.cgi?acc=GSE283136]The source data generated in this study are provided in the Supplementary Information (Supplementary Information 7Table 5) and the Source Data file. The genetic constructs are available via Addgene (#254460-254465). The processed data generated in this study have been deposited in the Zenodo database under accession code 19114282 [https://doi.org/10.5281/zenodo.19114282].

Research involving human participants, their data, or biological material

Policy information about studies with human participants or human data. See also policy information about sex, gender (identity/presentation), and sexual orientation and race, ethnicity and racism.

Reporting on sex and gender	SCTi003-A and MRIi003-A female, WTC11 male
Reporting on race, ethnicity, or other socially relevant groupings	not applicable
Population characteristics	not applicable
Recruitment	not applicable
Ethics oversight	not applicable

Note that full information on the approval of the study protocol must also be provided in the manuscript.

Field-specific reporting

Please select the one below that is the best fit for your research. If you are not sure, read the appropriate sections before making your selection.

[x] Life sciences [ ] Behavioural & social sciences [ ] Ecological, evolutionary & environmental sciences

For a reference copy of the document with all sections, see nature.com/documents/nr-reporting-summary-flat.pdf

Life sciences study design

All studies must disclose on these points even when the disclosure is negative.

Sample size	The standard sample size is n=3, for some instances we chose n=2 or n=1, which are clearly stated in the caption.
Data exclusions	We excluded data points from RT-qPCR analysis in Supplementary Figure 18, due to unspecific amplification, which was detected via melting curve analysis.
Replication	All experiments were conducted with at least three biological independent replicates.
Randomization	not applicable
Blinding	Blinding did not apply, we minimized pipetting error by using multichannel pipettes and mastermixes if possible.

Reporting for specific materials, systems and methods

We require information from authors about some types of materials, experimental systems and methods used in many studies. Here, indicate whether each material, system or method listed is relevant to your study. If you are not sure if a list item applies to your research, read the appropriate section before selecting a response.

Materials & experimental systems

n/a	Involved in the study	
[ ]	[x]	Antibodies
[ ]	[x]	Eukaryotic cell lines
[x]	[ ]	Palaeontology and archaeology
[x]	[ ]	Animals and other organisms
[x]	[ ]	Clinical data
[x]	[ ]	Dual use research of concern
[x]	[ ]	Plants

Methods

n/a	Involved in the study	
[x]	[ ]	ChIP-seq
[ ]	[x]	Flow cytometry
[x]	[ ]	MRI-based neuroimaging

Antibodies

Antibodies used	PAX-6 Antibody, anti-human, APC, REAfinity™, Sox2 Antibody, anti-human/mouse, FITC, REAfinity™, Sox17 Antibody, anti-human, APC, REAfinity™, CD184 (CXCR4) Antibody, anti-human, Vio® Bright FITC, REAfinity™, CD144 (VE-Cadherin) Antibody, anti-human, FITC, REAfinity™, CD140b Antibody, anti-human, APC, REAfinity™ (all Miltenyi)
Validation	*Describe the validation of each primary antibody for the species and application, noting any validation statements on the manufacturer's website, relevant citations, antibody profiles in online databases, or data provided in the manuscript.*

Eukaryotic cell lines

Policy information about cell lines and Sex and Gender in Research

Cell line source(s)	HEK293T (human), Neuro2a (murine), primary neurons (murine), hiPSC SCTi003-A (STEMCELL Technologies), hiPSC MRIi003-A, hiPSC WTC11
Authentication	HEK293T and Neuro-2a were bought from ECACC and were not additionally authenticated. Pluripotency of hiPSCs was authenticated by stainings, RTqPCR and NGS analysis.
Mycoplasma contamination	All cell lines were tested for mycoplasma contamination using MycoAlertTM Mycoplasma Detection Kit (LT07-318, Lonza). In addition, all cell lines are tested every 3 months for contamination by Hoechst 3334, which visualizes extranuclear speckles in case of a contamination.
Commonly misidentified lines (See ICLAC register)	not applicable

Plants

Seed stocks	not applicable
Novel plant genotypes	not applicable
Authentication	not applicable

Flow Cytometry

Plots

Confirm that:

- [x] The axis labels state the marker and fluorochrome used (e.g. CD4-FITC).
- [x] The axis scales are clearly visible. Include numbers along axes only for bottom left plot of group (a 'group' is an analysis of identical markers).
- [x] All plots are contour plots with outliers or pseudocolor plots.
- [x] A numerical value for number of cells or percentage (with statistics) is provided.

Methodology

Sample preparation	Cells were detached with accutase solution, spun down (300g, 3min) and resuspended in 10% formalin for fixation. After 10 min incubation on ice, cells were washed with PBS and resuspended in PBS + 2% FCS for FACS analysis.
Instrument	BD Symphony A3
Software	BD FACSDiva Software (BD Biosciences), FlowJo_v10.10.0
Cell population abundance	For FACS analysis, at least 10,000 events were recorded per condition.
Gating strategy	Cells were first gated based on forward scatter area (FSC-A) and side scatter area (SSC-A) to exclude debris (P1). Single cells were then selected using forward scatter height versus forward scatter area (FSC-H vs FSC-A) to exclude doublets and aggregates (P2). Green- and red-fluorescent populations were subsequently identified based on fluorescence intensity thresholds (P3).

- [x] Tick this box to confirm that a figure exemplifying the gating strategy is provided in the Supplementary Information.
